# Editorial: Engineered tissues using bioactive hydrogels

**DOI:** 10.3389/fbioe.2022.975907

**Published:** 2022-07-22

**Authors:** Miao Qin, Yi Deng, Sushila Maharjan, Zongliang Wang, Di Huang

**Affiliations:** ^1^ Research Center for Nano-Biomaterials and Regenerative Medicine, College of Biomedical Engineering, Taiyuan University of Technology, Taiyuan, China; ^2^ State Key Laboratory of Polymer Materials Engineering, School of Chemical Engineering, Sichuan University, Chengdu, China; ^3^ Division of Engineering in Medicine, Department of Medicine, Brigham and Women’s Hospital and Harvard Medical School, Boston, MA, United States; ^4^ Key Laboratory of Polymer Ecomaterials, Changchun Institute of Applied Chemistry, Chinese Academy of Sciences, Changchun, China

**Keywords:** tissue engineering, extracellular matrix, 3D structure, bioactive, hydrogels

Tissue damage, dysfunction and organ failure remain great challenges to cure, which have been the major causes of human morbidity and mortality. According to studies, the global cost associated with organ dysfunction and failure has been projected to hundreds of billions of dollars every year. Therefore, the regeneration of human tissues and organs have been widely explored to repair or replace defective tissues and organs while restoring their functions, thereby improving health and life quality. Several technologies including surgical reconstruction, organ transplant, *in situ* induced regeneration and tissue engineering have been employed to achieve regeneration of tissues and organs. Among these approaches, tissue engineering holds the promising approach to engineer various tissues and organs with the potential to meet the future needs of patients requiring the repair, replace and regeneration of tissues and organs.

Hydrogels have been proven to be promising candidates in tissue engineering field due to the distinct three-dimensional network structure and excellent biocompatibility, which could provide extracellular matrix like microenvironments and stimulus response properties in human tissues and organs. Consequently, endeavor has been pursued on the investigation of hydrogels for mimicking human tissues such as bone, skin, muscle, and so on. However, the clinical applications of hydrogels are still hampered owing to the intrinsic characteristics including poor mechanical performances, low bioactivity, uncontrollable degradation process, which leaves a huge gap compared to real human tissues. Among these drawbacks, insufficient bioactivity is the primary obstacle to be overcome for researchers, which exert a significant influence on tissue regeneration. To address this thorny issue, plenty of strategies such as introducing cells and bioactive molecules into hydrogels have been conducted. Even though bioactivity of the hydrogels has been improved, there still remain a large number of challenges that restrict their application in clinics. A variety of factors, such as the loading efficiency of cells and bioactive molecules, the interaction between hydrogels and bioactive molecules, the biostability of hydrogel-based engineered tissues system, etc., should be considered in hydrogel-based engineered tissues before their clinical application.

In this Research Topic, we present a number of interesting studies related to the development of bioactive hydrogels for mimicking tissues to facilitate the repair and regeneration of defective tissues. Broadly, there are two approaches towards recreating engineered tissues *in vitro*. One is development and fabrication of bioactive hydrogels. For example, Sun et al. have designed a composite hydrogel with antibacterial property for skin repair. They have fabricated decellularized ECM-based bio-scaffolds to promote remodeling of skin tissues. Similarly, Wu et al. have constructed injectable small extracellular vesicles laden hydrogel for accelerating bone regeneration. While Xu et al. have developed hybrid aerogel scaffold for osteonecrosis repair, Yu et al. have synthesized bio-hydrogel cell delivery system for retarding intervertebral disc degeneration. Furthermore, Wang et al. have summarized the application of bioactive hydrogels in intrauterine adhesion treatment and Lv et al. have summarized new treatment ideas in spinal cord injuries using hydrogels.

The other approach is utilization of different technologies. For instance, Sun et al. have shown the promising prospects of exosome-laden hydrogels in bone repair and Xin et al. have introduced versatile design strategies of bioactive hydrogels against osteomyelitis. Furthermore, Liu et al. have demonstrated the merits of 3D printing technology-based hydrogel in bone repair and reconstruction. With the encouraging strategies and approaches, we believe that the new paradigm will be introduced into tissue engineering field using bioactive hydrogels, which will advance the engineering of tissues and organs *in vitro* for their use in clinics.

